# OP20 Incorporating External Information In Survival Extrapolation Using A Simplified Bayesian Approach

**DOI:** 10.1017/S0266462324000837

**Published:** 2025-01-07

**Authors:** David McConnell, Arthur White, Felicity Lamrock, Joan O’Callaghan, Laura McCullagh, Joy Leahy, Lea Trela-Larsen

## Abstract

**Introduction:**

Cost-effectiveness modeling often requires extrapolation of survival data from clinical trials over a long-term horizon. The choice of extrapolation method is often uncertain and can have a profound impact on the results. We propose a novel Bayesian approach towards incorporating external information (e.g., registry data or clinical opinion) into the extrapolation process as a means of reducing this uncertainty.

**Methods:**

Standard parametric survival curves are fitted to immature time-to-event data using maximum likelihood estimation (MLE). Separately, external information on expected cohort-level survival at a future time point is used to specify a prior probability distribution. These are combined to generate posterior distributions of survival curve extrapolations that simultaneously incorporate both observed data and external information. This is done using importance sampling and multivariate normal approximations of the likelihood and posterior distributions; it requires only summary model parameter estimates (and not patient-level data). We apply our method to analyze survival data from the KEYNOTE-426 trial of pembrolizumab+axitinib in advanced renal cell carcinoma.

**Results:**

The method was implemented in R, and outputs survival curve parameters that are compatible with cost-effectiveness models developed in other software (e.g., Microsoft Excel). In all examples considered, our method resulted in extrapolated survival predictions that were more closely aligned with the external information compared with the standard (MLE-based) approach. Incorporation of external information decreased between-distribution variance (reduced structural uncertainty), and generally also decreased within-distribution variance as well (reduced parameter uncertainty). Results were comparable with those obtained from the method of Cooney and White, which uses similar ideas but requires full patient-level data and is more computationally complex.

**Conclusions:**

The extrapolation method we describe can reduce uncertainty when valid external information is available. Only MLE-based parameter estimates are required to implement our method, thus secondary model users such as HTA agencies can adjust survival extrapolations from existing cost-effectiveness models without access to patient-level data. Implementation is straightforward and computationally efficient, and outputs are easily incorporated into existing cost-effectiveness models.

